# Electron microscopy by specimen design: application to strain measurements

**DOI:** 10.1038/s41598-017-12695-8

**Published:** 2017-09-29

**Authors:** Nikolay Cherkashin, Thibaud Denneulin, Martin J. Hÿtch

**Affiliations:** CEMES, CNRS, 29 rue Jeanne Marvig, 31055 TOULOUSE, Cedex 4, France

## Abstract

A bewildering number of techniques have been developed for transmission electron microscopy (TEM), involving the use of ever more complex combinations of lens configurations, apertures and detector geometries. In parallel, the developments in the field of ion beam instruments have modernized sample preparation and enabled the preparation of various types of materials. However, the desired final specimen geometry is always almost the same: a thin foil of uniform thickness. Here we will show that judicious design of specimen geometry can make all the difference and that experiments can be carried out on the most basic electron microscope and in the usual imaging modes. We propose two sample preparation methods that allow the formation of controlled moiré patterns for general monocrystalline structures in cross-section and at specific sites. We developed moiré image treatment algorithms using an absolute correction of projection lens distortions of a TEM that allows strain measurements and mapping with a nanometer resolution and 10^−4^ precision. Imaging and diffraction techniques in other fields may in turn benefit from this technique in perspective.

## Introduction

Developments in transmission electron microscopy (TEM) have centered on improving instrumentation and optical configurations. Considerable effort has also been put into developing sophisticated specimen preparation methods and equipment, from the humble ion-beam polisher, through tripod polishing and ultramicrotomy, to multifunction focused-ion beam (FIB) systems. However, the desired final specimen geometry is always the same: a thin foil of uniform thickness. Here we will show that judicious design of specimen geometry can make the all the difference and that experiments can be carried out on the most basic electron microscope and in the usual imaging modes.

The example we have chosen to illustrate electron microscopy by specimen design is the measurement of strain in thin-films and devices. Strain engineering is now an important feature of many research areas, from strained-silicon transistors^1^ to ferroelectrics^2^ and thermoelectric materials^3^. It is therefore not surprising that many TEM techniques have been developed over the years to measure it accurately^4^. These include quantitative analysis of high-resolution transmission electron microscope images (HRTEM)^5–7^, convergent-beam electron diffraction (CBED)^8^, nano-beam electron diffraction (NBED)^9,10^, dark-field off-axis electron holography (DFEH)^11^, dark-field inline holography (DIH)^12,13^ and more recently STEM moiré fringes (SMF)^14,15^.

All these techniques have specific instrumental requirements such as aberration correctors, Lorentz lenses or electrostatic biprisms (for holography). Even DIH and CBED require an imaging energy filter faced with the complexity of the data simulation and interpretation^12,13,16,17^. Our aim is not to belittle instrumental developments but to explore an alternative route to making measurements with electron microscopy that can, in some ways, be superior to existing techniques. How then can we design the specimen geometry to perform the technique on the most basic conventional TEM: by returning to the moiré imaging phenomenon known from the very beginnings of electron microscopy^18,19^.

**Figure 1:**
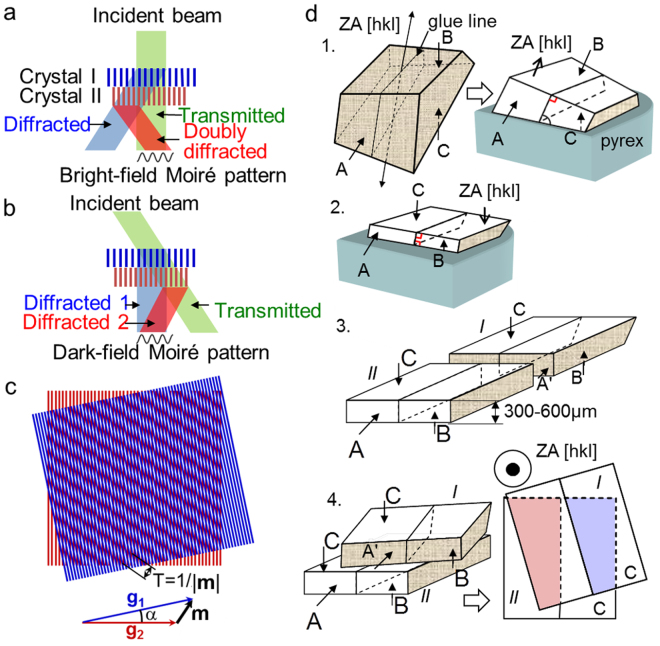
Formation of moiré fringes: two superimposed crystals with slightly different lattice parameters and rotated by an angle, α, around the axis of the electron beam create interference: (**a**) In bright-field, between transmitted beam and a doubly diffracted beam. (**b)** In dark-field, between two diffracted beams. (**c**) Geometry of moiré fringe formation. (**d**) Four principle stages of the tripod based preparation method of double lamella cross-sectional sample:1. First surface (B) polishing parallel to zone axis [hkl]; 2. Second surface (C) polishing parallel to B; 3. Cutting into two pieces, 4. Two lamellas superposition.

Moiré patterns are a general interference phenomena that appear when two periodic arrays are superposed^20^. In TEM, they usually occur by chance, when two crystals with slightly different lattice parameters or orientation are superposed along the path of the electron beam (Fig. 1). In a bright-field (BF) image, the fringe pattern results from the interference of the transmitted beam passing through the two crystals with a doubly diffracted beam in crystal II. In a dark-field (DF) image, the resulting Moiré pattern arises from an interference between two beams diffracted in crystal I and II. Moiré fringes are formed if there is a vector difference ${\bf{m}}={{\bf{g}}}_{2}-{{\bf{g}}}_{1}$ between the diffraction vectors I, **g**_1_ and II, **g**_2_. The moiré spacing, m, can therefore be linked to the strain, or orientation changes, in the sample with the spatial resolution of the measurements limited to the moiré spacing. As we will show (see Methods), the corresponding lattice distortions can be conveniently extracted from moiré patterns by using the formalism of geometric phase analysis (GPA)^18^.

Happenstance has allowed moiré patterns to be investigated in different epitaxial samples, such as islands of InAs on GaAs^21^, Ge islands on Si^22^ and YBa_2_Cu_3_O_7_ thin film on MgO^23^ where in plan-view the substrate is naturally superposed with the region of interest. The geometry is fixed and there can be no choice of orientation or fringe spacing. Here, we proposed two sample preparation methods that allow the formation of controlled moiré patterns, for more general structures, in cross-section and at specific sites. We will show how the spatial resolution of strain maps can be chosen as a function of the specimen preparation.

The principal technique is based on tripod specimen preparation (Fig. 1d). A cross-sectional lamella is prepared in the desired zone axis and carefully cut into two pieces that are put one upon the other. The upper lamella is displaced laterally until the substrate is superposed with the region of interest and rotated (if necessary). The art relies in precisely controlling the parallelism of the two adjacent surfaces during superimposition and the in-plane rotation angle that determines the spatial resolution of the technique. The possible disorientation of 0.05° between the two adjacent surfaces (see Methods) is one order of magnitude smaller than the values of typical Bragg angles varying in the range of 0.3–0.6°. Thus, the zone of interest can be considered as being in the same orientation as the reference lattice. A set of examples will illustrate the technique using a conventional TEM (a JEOL 2010 operating at 200 kV).

The first example consists of five fully relaxed Si_1−x_Ge_x_ layers grown on top of a polished Si_0.65_Ge_0.35_ virtual substrate^24^. This sample which has been characterized by X-ray diffraction (XRD) and Secondary Ion Mass Spectroscopy (SIMS)^25^ is ideal to show the accuracy and precision of the moiré-sample technique. In addition, the layers are already fully relaxed in the bulk sample, so there will be no thin-film relaxation in the TEM lamellas.

**Figure 2:**
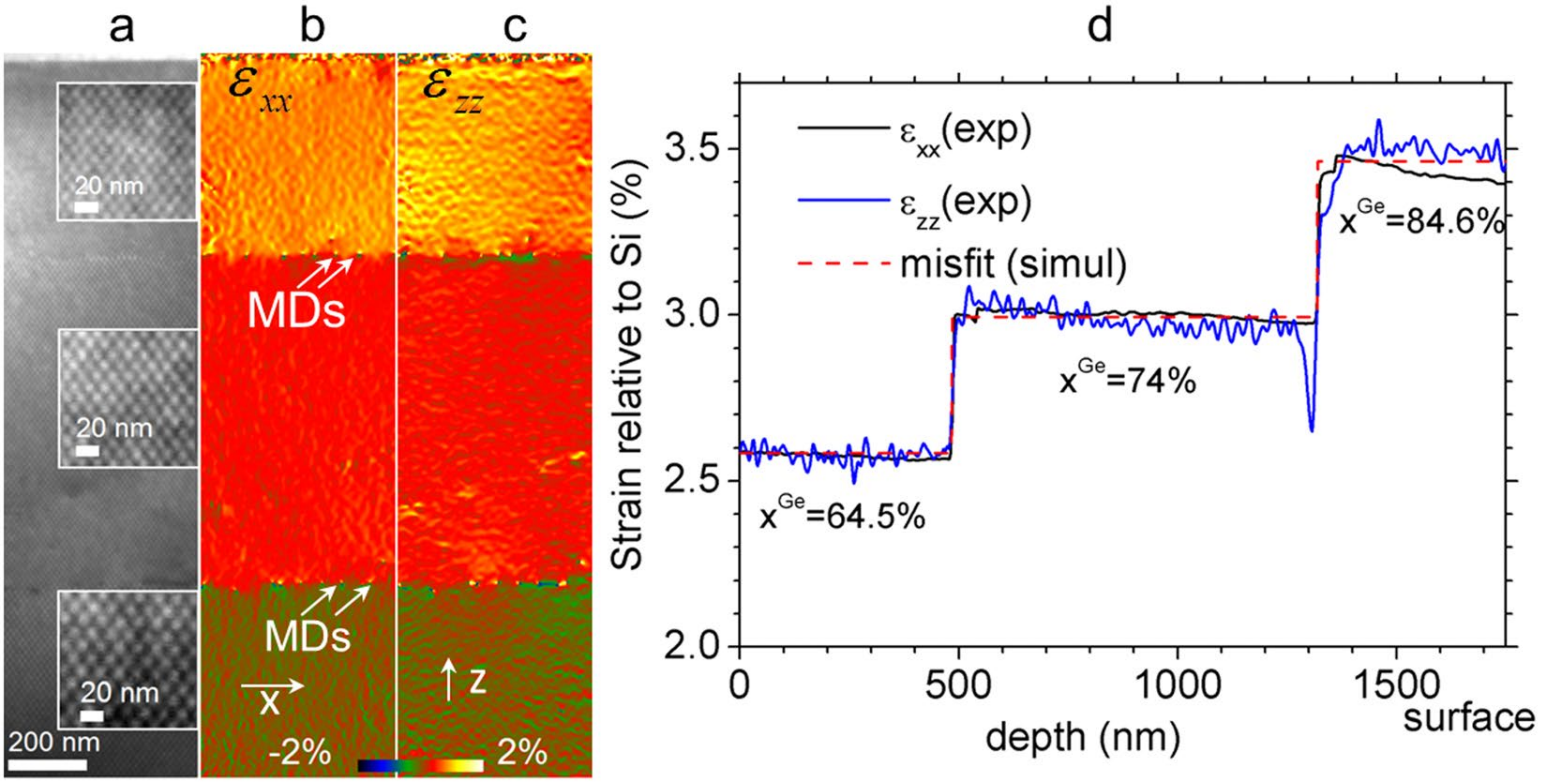
Structure consisting of five high Ge content completely relaxed Si_1−x_Ge_x_ layers with x = 45%, 52%, 64.5%, 7 4% and 85% grown on top of a polished Si_0.65_Ge_0.35_ virtual substrate: (**a**) Bright-field cross-sectional (1–10) zone axis (ZA) [1–10] Moiré dot pattern formed over three upper layers (inserts show the zoomed parts of each layer) of a double lamella sample prepared with 0° rotation between two lamellas. (**b**) In-plane ${\varepsilon }_{xx}^{S{i}_{0.355}{e}_{0.645}}$ and c, Out-of-plane ${\varepsilon }_{zz}^{S{i}_{0.355}{e}_{0.645}}$ stain maps. Misfit dislocations (MDs) are shown by arrows in b. (**d**) Experimental strain profiles (black and blue lines) and simulated misfit profile (red dashed line) calibrated with respect to Si.

The moiré sample was prepared in (1–10) orientation, without any rotation between the two lamellas. A double moiré contrast is then formed by the interference of the two sets of (111) lattice planes of the Si substrate and the Si_1−x_Ge_x_ layers (see insert in Fig. 2a). The geometric phase of the two sets of moiré fringes was determined and corrected for the projector lens distortions^26^. Given that the moiré fringe spacing was 12.5 ± 0.1 nm in the bottom layer (the error is given by the magnification calibration accuracy of 1%), a spatial resolution of 25 nm was obtained for the strain analysis. The resulting strain (more precisely, deformation) maps give the difference between the local lattice parameter and the Si substrate in the x-direction parallel to the interface, and in the z-direction of growth (Fig 2b and c respectively).

The layers being fully relaxed means that there should be no difference between the strain in the bulk sample and the TEM lamellas and the measurements are therefore directly comparable with X-ray data. Figure 2d shows vertical intensity profiles of ${\varepsilon }_{xx}$(black line) and ${\varepsilon }_{zz}$(blue line) averaged over a 400 nm distance along the interfaces which were recalibrated with respect to Si. The values of deformation measured both parallel and perpendicular to the layers, ${\varepsilon }_{xx}$ and ${\varepsilon }_{zz}$, are in very good agreement with the previously determined misfit values (summarized in Table 1) for x = 64.5%, 74% and 84.6%^23^. We can estimate the accuracy of the technique to be 0.02% and the precision from the standard deviation of the strain, to be as high as 0.01% for ${\varepsilon }_{xx}$ and 0.02% for ${\varepsilon }_{zz}$.

**Table 1:** Compilation of the results from the fully-relaxed Si_1−x_Ge_x_ multilayers.

Ge composition, *x*	Lattice parameter, ${a}^{S{i}_{1-x}G{e}_{x}}$	Mismatch, $\delta$	Measurements	
			${\varepsilon }_{xx}$	*ε*_*zz*_
64.5%	0.5571 nm	2.58%	2.57 ± 0.09%	2.57 ± 0.11%
74.0%	0.5594 nm	3.00%	2.99 ± 0.06%	2.96 ± 0.08%
84.6%	0.5619 nm	3.46%	3.42 ± 0.07%	3.49 ± 0.12%

Expected lattice parameters given by ${a}^{S{i}_{1-x}G{e}_{x}}={a}^{Si}+0.02005{\rm{x}}+0.00263{{\rm{x}}}^{2}$ [29], ${a}^{Si}=0.5431\,nm$ and mismatch, $\delta =({a}^{S{i}_{1-x}G{e}_{x}}-{a}^{Si})/{a}^{Si}$. Measurements averaged over a 350 nm square area.

This example shows that strain maps can be obtained over a huge field of view, 500 nm by 2 microns, which is very difficult to obtain with existing techniques, to very high precision and accuracy. Indeed, the accuracy is only limited by the calibration of the magnification since the moiré fringes measure the difference in lattice parameter between the substrate (upper lamella) and the area of interest (lower lamella). Unlike GPA or DFEH, no reference region is required as this has been already supplied by the substrate lamella. The precision is determined by the contrast of the moiré fringes. The spatial resolution (25 nm) is low but details in a vicinity of the interfaces reveal even the presence of misfit dislocations at the interfaces (white arrows in Fig. 2b). We will show in the next example how the spatial resolution of the moiré sample technique can be adjusted to even nanometer resolution by applying controlled sample rotations during preparation.

**Figure 3:**
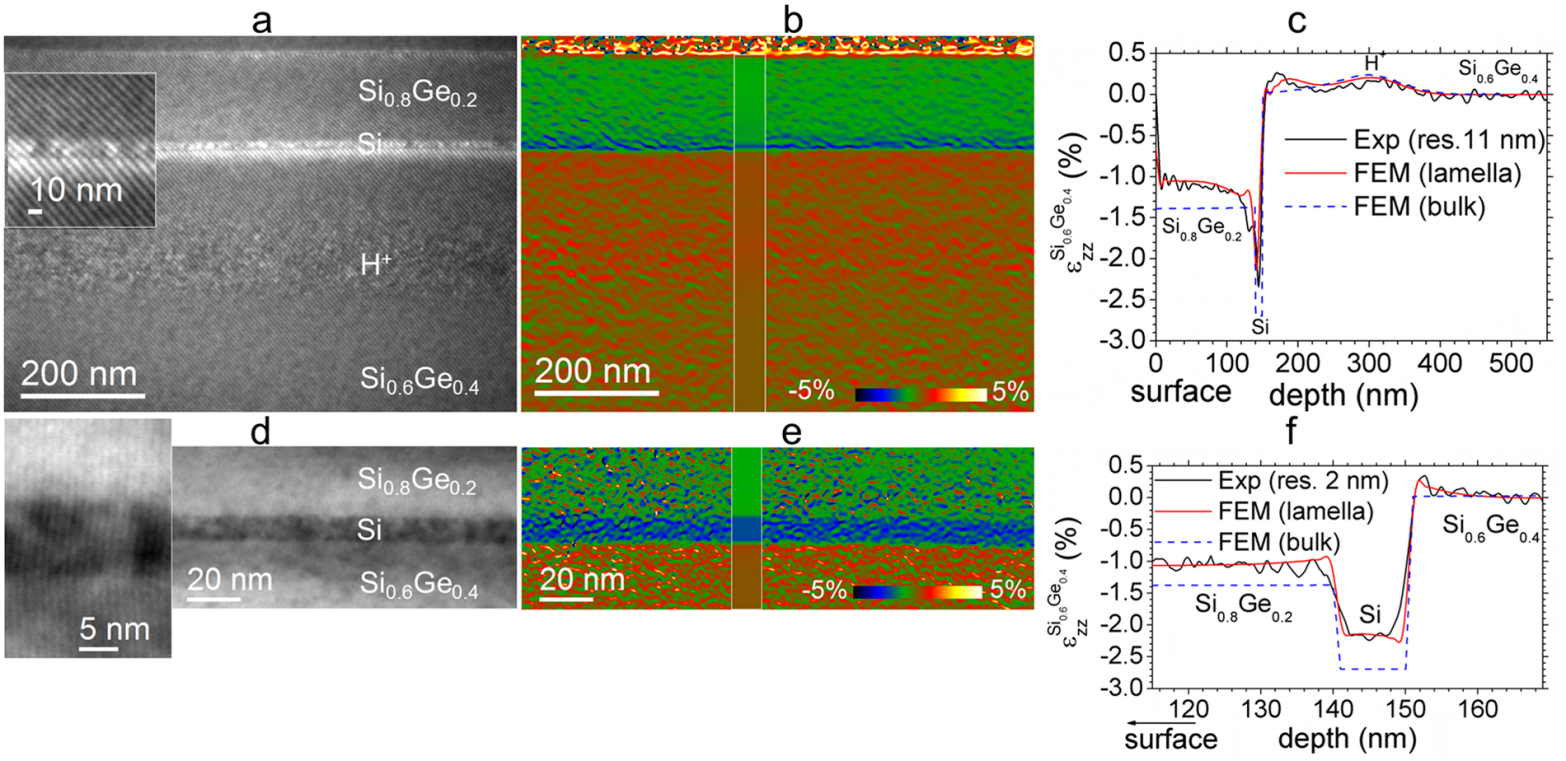
Structure consisting of a Si_1−x_Ge_x_ virtual substrate capped by a thick, constant composition completely relaxed Si_0.6_Ge_0.4_/10 nm-thick Si /170 nm-thick Si_0.8_Ge_0.2_ layers implanted by H^+^ ions. Cross-sectional (1–10) double lamella samples with (**a–c**), 3.2° (sample ‘A’) and (**d–f**) 7.8° (sample ‘B’) rotation between lamellas: dark-field single moiré fringe images taken with a, g = 111 and d, g = 004. (**b,e**) Corresponding out-of-plane ${\varepsilon }_{zz}^{S{i}_{0.6}G{e}_{0.4}}$ experimental strain maps with simulated strain maps inserted. (**c**,**f**), Line profiles: experimental strain profiles (black lines), FEM simulated in a TEM lamella (red lines) and in bulk (blue dashed lines).

The example structure consists of a Si_1−x_Ge_x_ virtual substrate capped by a thick, constant composition completely relaxed Si_0.6_Ge_0.4_/10 nm-thick Si/170 nm-thick Si_0.8_Ge_0.2_ layers implanted by H^+^ ions (see Methods). Two different moiré samples ‘A’ and ‘B’ were prepared in (1–10) cross-section, overlapping the Si substrate with the region of interest, as before, but this time with applying rotations of 3.2° (sample ‘A’) and 7.8° (sample ‘B’) between the upper and lower lamellas. Figure 3a–c show a dark-field moiré image taken with g = 111, out-of-plane strain map extracted with a spatial resolution of 11 nm and corresponding strain profile (black line) of the sample ‘A’. Whilst a spatial resolution of 11 nm is good enough for mapping strain in thick Si_1−x_Ge_x_ layers over a large field of view, it blurs the strain profile in the thin Si layer.

Figure 3d shows a DF image of the second sample taken with g = 004 demonstrating a Moiré pattern consisting of one set of fringes (zoomed part around the Si layer is shown in insert) with a period of only 1 nm. The map of ε_zz_ (Fig. 3e) was extracted with a 2 nm spatial resolution revealing the details of the strain in thin Si layer clearly.

Thin-film relaxation is significant in this sample, so FEM modelling was carried out assuming a foil thickness of 30 nm (see Methods). The deformation is reduced by about 22% with respect the values in a bulk structure (Fig. 3f, blue dashed line). The excellent agreement with the experimental profiles suggests that an accuracy and precision of 0.1% can be reached even at such ultimate spatial resolution of 2 nm.

**Figure 4:**
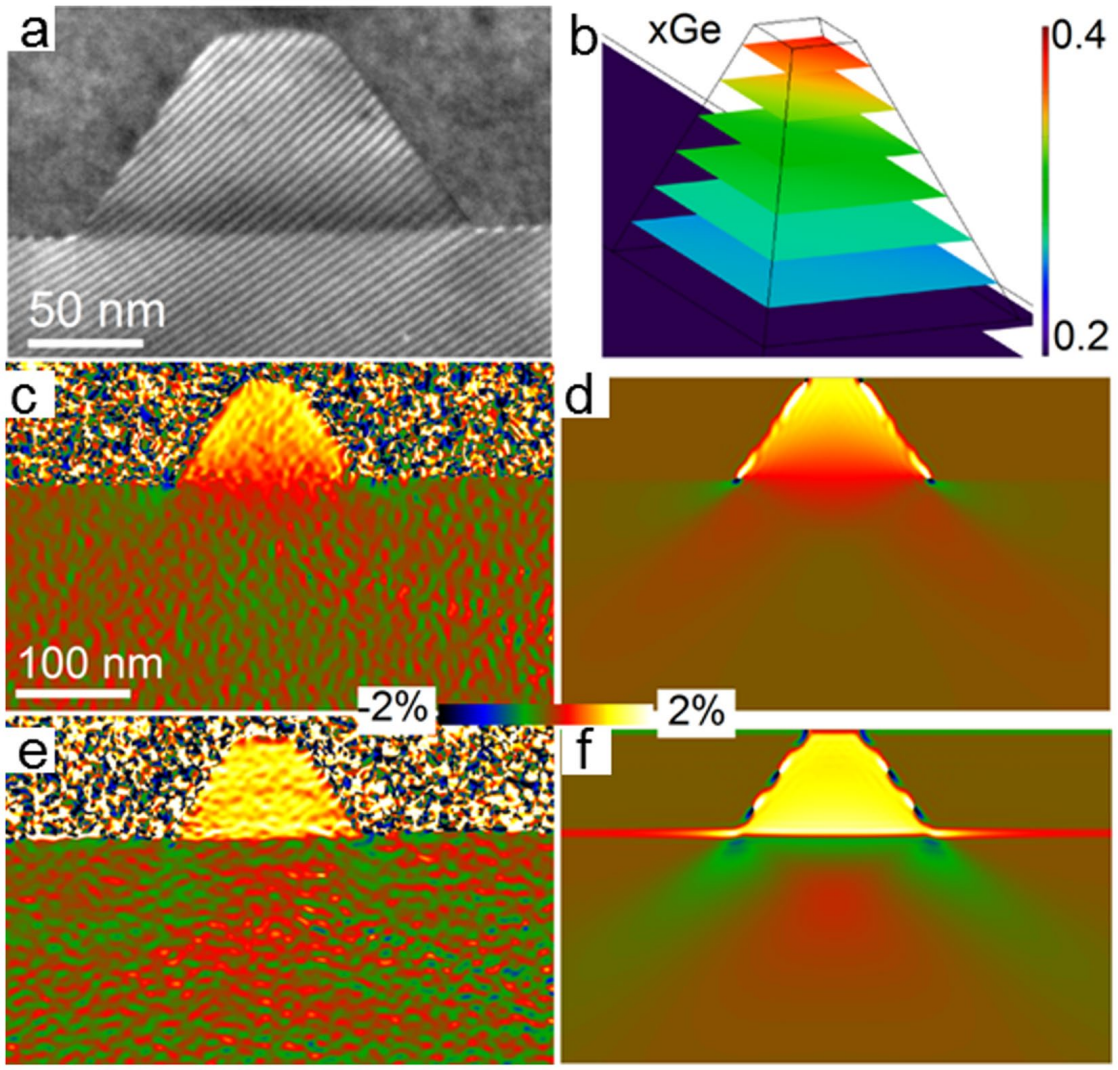
Moiré analysis of coherently strained SiGe quantum dot on Si substrate: (**a**) (111) moiré fringes. (**b**) Modelled germanium concentration. (**c,e**) Measured strain component parallel and perpendicular to interface, respectively. (**d,f**) Corresponding strain maps from finite element analysis.

**Figure 5:**
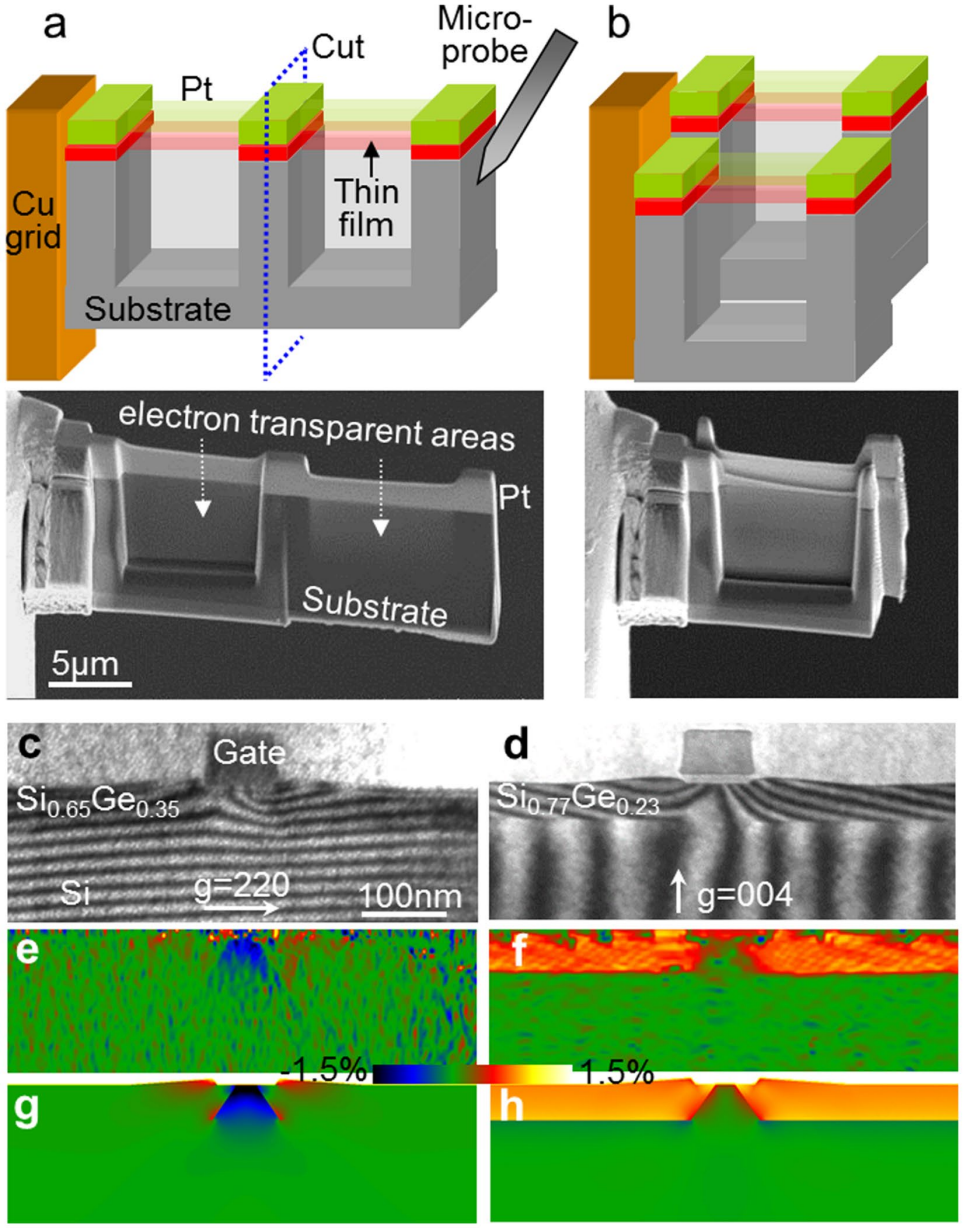
(**a**,**b**) Principle stages of the FIB based preparation method of double lamella cross-sectional sample: (**a**) Schematic representation and SEM image of an epitaxial sample prepared by focused ion beam. A piece of sample was cut from the bulk, lifted-out and attached to a grid. Two windows were thinned to electron transparency. (**b**) Schematic representation and SEM image of the finished double lamella sample. Right window in (**a)** was freed and moved behind the left one using a micro-probe. It was shifted of a few microns in the vertical direction such a way that its substrate overlaps the epitaxial thin film of the fixed window. (**c–h**) Site-specific moiré analysis of strained-silicon transistor: (**c,d**) Moiré fringes of two similar transistors for (220) and (004) lattice planes, respectively. (**e,f**) Measured strain components, respectively parallel and perpendicular to interface. (**g,h**) Corresponding strain maps from finite element analysis.

The tripod method is well efficient for mapping inhomogeneous strain fields associated to 3D objects such as quantum dots (Fig. 4a,c,e) in excellent quantitative agreement with 3D FEM simulations (Fig. 4b,d,f). However, devices usually require a site-specific method of preparation. For this reason, we have also developed a moiré-sample preparation method with the FIB (Fig. 5).

The rotation angle, and tilt, between the two lamellas is much more difficult to control in the FIB than for the conventional specimen preparation. For example, only one set of moiré fringes were available for each sample and the fringe spacing, notably for the (004) lattice planes, could have been finer. Nevertheless, this example shows that FIB preparation is a possibility to explore for strain mapping where site specificity is necessary. We believe that a further development of the FIB method would allow to superimpose lamellas with a controlled angle of rotation and, thus, to reach a nanometer resolution for strain mapping.

Strain measurements by specimen design are not only comparable with existing techniques but can be obtained for fields of view only limited by the sample dimensions. Strain is also measured with respect to a well-defined reference ensuring high accuracy. We anticipate that the principle of specimen design can be extended to other types of measurement and to other types of epitaxial samples such as ferroelectric oxides, metals and ceramics.

## Methods

### Sample preparation

The growth of the fully relaxed Si_1−x_Ge_x_ layers is fully described in^24^, the Si_1−x_Ge_x_ quantum dots in^27^, and the MOSFET devices in^28^. The H^+^ implanted structure consisted of the following layers grown by CVD on a Si_1−x_Ge_x_ virtual substrate: a thick layer of completely relaxed Si_0.6_Ge_0.4_, 10 nm-thick in-plane tensile strained Si, and 170 nm-thick in-plane tensile strained Si_0.8_Ge_0.2_ layer. The sample was then implanted by H^+^ ions at 30 keV to a fluence of 1 × 10^16^ cm^−2^ in the same way as for the silicon sample described in^29^.

### Moiré sample preparation

The four principle stages of the tripod preparation method are (Fig. 1d): (1) gluing of a cross-sectional sample, cutting a slice, tripod based grinding of the slice butt (A), grinding and polishing of the slice front side (B); (2) grinding and polishing of the slice back side (C) leaving a 300 µm–600 µm-thick lamella with mutually parallel B and C surfaces. Both surfaces B and C should be approximately perpendicular to the desired direction of the sample observation [hkl] (marked as zone axis (ZA) [hkl]). Polishing is realized on a felt using a colloidal solution of the silica grains to atomically smooth surfaces B and C; (3) cutting of the slice into 2 quasi-equivalent pieces I and II; (4) superposition of two pieces in a manner that: the surface C of part II is face to face with respect to surface B of part I; the surface A’ of part I is above the surface A of part II; part I is displaced at a distance of 400–600 µm from the boards of part II; part I is rotated with respect to part II at the desired angle (between 0–15°) in the plane of surface B. The surfaces B and C become parallel when the polished slice gets the same thickness over its whole length and width. The thickness of the slice is controlled in an optical microscope with a ± 5 µm precision. For a 6 mm long slice, such thickness variation corresponds to about 0.05° disorientation between the surfaces B and C.

The sample is put in a press, epoxy glue applied around the edges, and then into an oven at 80 °C. The mechanical pressure and the epoxy glue are necessary to have a good contact between the surfaces, to minimize the distance between them, if any, and to immobilise the configuration. Both parts I and II of the double lamella are then thinned by tripod grinding down to a thickness of 10 µm. The final stage comprised of Ar^+^ ion-beam thinning on both sides of the sample with a PIPS (Gatan) until electron transparency is obtained. As a result, the zones of interest in part II are covered by the substrate (part I) consisting of known and undistorted material (red zone, step 4) or vice versa (blue zone, step 4).

The FIB samples (Fig. 5) were prepared using a Helios FIB-SEM (FEI). A standard extraction procedure was first carried out as follows. A 200 nm thick Pt layer was deposited with electron beam assistance to protect the sample surface, followed by ion-beam deposition of a thick 3 μm layer of Pt. Trenches were milled on both sides of the deposit. A piece of sample was then cut and lifted out using a nano-manipulator (Omniprobe). The piece was attached to a grid using Pt deposition and thinned down to about 500 nm at 30 kV. Two 8 μm wide windows were then thinned to electron transparency (Fig. 5a,b) at 8 kV to reduce the amorphization of the surfaces. A tilt of ±2° was applied to compensate for the Gaussian shape of the ion beam.

The probe was then attached again to the free side of the sample and the two windows were separated (Fig. 5c). The detached window was positioned behind the other one and shifted a few microns in the vertical direction, in such a way that its substrate overlaps the epitaxial thin film of the fixed window. For the last steps, care should be taken to avoid imaging the lamellae in order to avoid re-deposition of Pt on the surfaces. Since the alignment of the lamellas with the micro-probe is never perfect, small rotations occur when detaching/attaching the lamella. The rotation angle between the two lamellas is typically less than 1° which corresponds to a moiré period greater than 20 nm.

### TEM observations

The moiré samples prepared by tripod polishing were observed on a Jeol 2010 TEM with a LaB_6_ gun operating at 200 kV. Images were acquired on a CDD camera (1376 × 1032 pixels) using 1 to 5 s acquisition times. The FIB-prepared samples were observed on a Philips CM20FEG microscope, equipped with a Schottky field emission gun operated at 200 kV. Images were acquired on a CCD camera (2k by 2k pixels binned by 2) using 5 to 10 s acquisition times.

### Moiré analysis

Moiré patterns were analyzed using modified version of HoloDark 1.0 (HREM Research, Inc.), plugin for the image processing software Digital Micrograph (Gatan Inc.). The phase of the moiré fringes was assumed to be equal to the geometric phase of the diffracted beams forming the moiré pattern and the strain maps calculated accordingly. The geometric phase was corrected for the projector lens distortions^26^. The spatial resolution for the strain measurements was defined by the radius of the mask used for inverse Fourier reconstruction and was, at best, twice the moiré period.

For the reconstruction of the geometric phase for the FIB-prepared samples, where the fringe spacing is large, we used a reconstruction method functioning in real space^30^ which allows the improvement of the spatial resolution by a factor of 2 with respect to the Fourier method used in GPA^6^ or DFEH^11^. It involves several steps: detection of the fringe minimums and maximums, calculation of envelope functions, normalization of the interference pattern to retrieve the cosine function and finally calculation of the phase image.

### Modeling

Finite element method (FEM) modeling was performed using the structural mechanics module of COMSOL Multiphysics. The samples were simulated in 2D (when appropriate) and 3D, taking into account the elastic relaxation at the free surfaces of the thin foil. Mismatch between epitaxial layers was incorporated in the usual way as an initial deformation. The deformation and displacement fields were averaged over the thickness of the lamella using a weighting function^31,32^ and imported into Digital Micrograph (Gatan Inc.). Moiré patterns and phase images were then calculated from the simulated displacement field and treated exactly as the experimental images. The foil thickness was measured using the extinction fringes of {111} reflection of the crystal in dark-field TEM imaging.

The modeling of the H^+^ ions implanted sample required a specific treatment, as described in^29,33^ for a silicon sample implanted with H^+^ ions under similar conditions. First the H profile, ${c}_{H}(z)$, as a function of the depth, z, was determined by SRIM code^34^. The misfit was described analytically as ${\varepsilon }_{xx}(z)\cong -0.17{c}_{H}(z)$. A weighting function was calculated for a TEM lamella thickness of 100 nm, g = 111, deviation from exact Bragg conditions s = 0.012 nm^−1^.

For the quantum dot sample, the Ge composition distribution within the island was modeled in the same way as for a pyramid-shaped InGaAs/GaAs quantum dot^35^ but modified for a 45° rotation of the pyramid square base (Fig. 4b). The concentration of Ge in the pyramid of height, H, and base length, L, is described by $c(x,y,z)={c}_{base}+\frac{Lz({c}_{facet}(z)-{c}_{base})}{H(L-x-y)}$ where the origin (0,0,0) is situated at the center of the island base and the Ge distribution along the facets is given by ${c}_{facet}(z)={c}_{facetbase}+({c}_{apex}-{c}_{facetbase})\sqrt{z/{H}_{pyr}}$. The constants ${c}_{base}=0.23$,$\,{c}_{facetbase}=0.15$ and ${c}_{apex}=0.39$ stand for the Ge concentration in the base of the object and the facets for *z *= 0 and for the Ge concentration in the apex of the full pyramid for $z={H}_{pyr}$.
